# Comparison of classical Fabry and its p.D313Y and p.A143T variants by cardiac T1 mapping, LGE and feature tracking myocardial strain

**DOI:** 10.1038/s41598-023-32464-0

**Published:** 2023-04-10

**Authors:** Maxim Avanesov, Anahid Asgari, Nicole Muschol, Anja Friederike Köhn, Enver Tahir, Gerhard Adam, Paulus Kirchhof, Gunnar Lund, Ersin Cavus, Monica Patten

**Affiliations:** 1 Department of Diagnostic and Interventional Radiology and Nuclear Medicine, University Hospital Hamburg Eppendorf, Hamburg, Germany; 2 Department of Pediatrics, International Center for Lysosomal Disorders (ICLD), University Medical Center Hamburg-Eppendorf, Hamburg, Germany; 3 Department of Cardiology, University Heart and Vascular Center Hamburg Eppendorf, University Hospital Hamburg Eppendorf, 20246 Hamburg, Germany; 4 Deutsches Zentrum Für Herz-Kreislauf-Forschung e.V. (German Center for Cardiovascular Research), Partner Site Hamburg/Kiel/Lübeck, Germany, Hamburg, Germany

**Keywords:** Cardiology, Cardiac hypertrophy

## Abstract

Cardiac manifestation of classical Fabry disease (cFD) varies with sex and presence of left ventricular hypertrophy. p.D313Y/p.A143T variants (vFD) represent milder late-onset phenotypes, however, data on vFD are scarce. Patients with FD (cFD = 37;vFD = 14) and 14 healthy controls underwent 1.5 T CMR including Cine, LGE, native T1 mapping(nT1) and myocardial strain(CMR-FT). CMR-FT was assessed using ventricular longitudinal, circumferential, radial (LV-GLS/RV-GLS, LV-GCS/LV-GRS), and atrial longitudinal strain (LA/RA_Total_, LA/RA_Conduit_, LA/RA_Booster_). In cFD reduced myocardial strain (LV-GLS: −20 ± 4 vs. −24 ± 3%,*p* = 0.007; LV-GCS: −20 ± 4 vs. −26 ± 4%,*p* = 0.002, LA _Total_ -GLS: 29 ± 10 vs. 37 ± 6%,*p* = 0.007; LA _Conduit_ -GLS: 15 ± 10 vs. 23 ± 5%,*p* = 0.003) and nT1 values (951 ± 51 ms vs. 1036 ± 20 ms, *p* < 0.001) were observed compared to controls. In vFD findings were comparable to controls. LV-GCS provided the closest Area under the curve (AUC) to nT1 (0.84 vs. 0.92, *p* > 0.05) for discrimination of cFD versus controls. Significantly lower LV-GLS/LV-GCS was found in male compared to female cFD (−19 ± 4 vs. −22 ± 4%, *p* = 0.03). In six non-hypertrophied female cFD with normal nT1 LA_Total_ -GLS was the only discriminating parameter with an accuracy of 86%. LV-GLS, LV-GCS and LA_Total_ -GLS can detect impaired cardiac mechanics of cFD besides nT1. LA_Total_ -GLS might identify non-hypertrophied female cFD. Variants p.D313Y/p.A143T did not reveal cardiac involvement by multiparametric CMR.

## Introduction

Fabry disease (FD) is a rare X-linked, lysosomal storage disease caused by mutations in the alpha galactosidase A gene (GLA) leading to a deficiency of its encoding lysosomal enzyme^1^. Progressive GLA substrate accumulation, especially the sphingolipid globotriaosylceramide (Gb3) in the heart^2^ causes left ventricular hypertrophy (LVH), myocardial fibrosis and arrhythmias^2^ up to lethal FD cardiomyopathy^3^.

Besides the classical phenotype of FD, milder late-onset phenotypes and genetic variants of unknown significance have been described previously^4^. p.D313Y and the p.A143T variants are novel *GLA* mutations, caused by substitutions of tyrosine for aspartic acid at codon 313 and alanine for threonine at codon 143 of the GLA protein, respectively. Patients with these mutations (vFD) were more often oligosymptomatic compared to classical FD patients (cFD), however with apparent cardiac manifestations^5–7^. In contrast to cFD, where enzyme replacement (ERT) or chaperone therapy may reduce LVH and improve myocardial function^8^, a treatment in vFD is controversially discussed^4,6^.

CMR is crucial for an accurate, non-invasive assessment of cardiac manifestations of FD. Late gadolinium enhancement (LGE) is present at progressed FD stage^9^ while native T1 reduction may visualize myocardial sphingolipid accumulation at earlier^10^ and pre-hypertrophic FD stages^11^. CMR feature tracking strain (CMR-FT) may assess early changes in myocardial mechanics more sensitive compared to echocardiography due to the excellent image quality across both ventricles^12^. However, the data on T1 mapping^13^ and CMR-FT analysis in FD is scarce and refer to cFD only^14,15^.

The purpose of the study was (i) to evaluate possible alterations of T1 mapping and CMR-FT in all four cardiac chambers in cFD and in patients carrying the p.D313Y or p.A143T variants (vFD) compared to controls and (ii) to compare the diagnostic yield of the multiparametric CMR parameters to identify Fabry patients.

## Materials and methods

### Study population

The study investigated 51 patients with genetically proven FD (cFD = 37^4^, vFD = 14^5^ (p.D313Y = 10; p.A143T = 4), S1, supplement). The control group included 14 healthy volunteers without previously known cardiac diseases, with normal cardiac biomarkers, a maximum septal wall thickness < 12 mm, no LGE, sex-dependent normal LVEF^16^, no cardiac medication, age ≥ 18 years.

All Fabry patients were outpatients in stable health condition, referring to a clinically indicated CMR between 12/2014 and 12/2017. Exclusion criteria included standard contraindications to CMR. All participants underwent CMR, ECG and echocardiography during the same study visit. Blood samples of all participants were obtained routinely ± 3 days before/after CMR. Troponin T, NTproBNP, and lyso-Gb3 were quantified. Troponin T concentrations ≥ 14 pg/mL were considered abnormal, using the 99th percentile of the normal distribution^17^. NT-proBNP concentrations > 125 pg/ml were considered abnormal^18^. Lyso-Gb3 concentrations > 0.9 ng/ml were considered abnormal, using the 95^th^ percentile of healthy individuals^19^. The study followed the principles outlined in the Declaration of Helsinki and was approved by the local ethics committee (State of Hamburg Chamber of Medical Practitioners). All patients and controls gave their written informed consent to use CMR information for research purposes.

### CMR protocol

Clinically indicated CMR was performed on a 1.5-T scanner (Achieva,Philips Medical Systems,Best, Netherlands). Standard retrospectively gated SSFP cine was performed in short- and in long-axis to assess the LV volumes and function. Imaging parameters were: voxel size 1.36 × 1.36 × 6 mm^3^, TE 1.67 ms, TR 3.34 ms, FA 60°, parallel acquisition technique: SENSE. LGE images in short- and in long-axis were acquired using end-diastolic phase-sensitive inversion recovery (PSIR) sequence ten minutes after bolus injection of contrast media (0.1 mmol/kg Gd-DOTA (Dotarem®) with following parameters: voxel size 1.36 × 1.36 × 8 mm^3^, TE 2.40 ms, TR 5.50 ms, FA 15°. Native T1 mapping was performed using a 3(3)5-modified Look-Locker inversion recovery (MOLLI) sequence on end-diastolic LV short axes in the septal LV wall at basal and mid cavity level, avoiding the blood–myocardial boundary^16^. Normal T1 values were within the range of the mean ± 2 SD of the mean T1 time of the control group^20^.

### CMR data analysis

**Figure 1:**
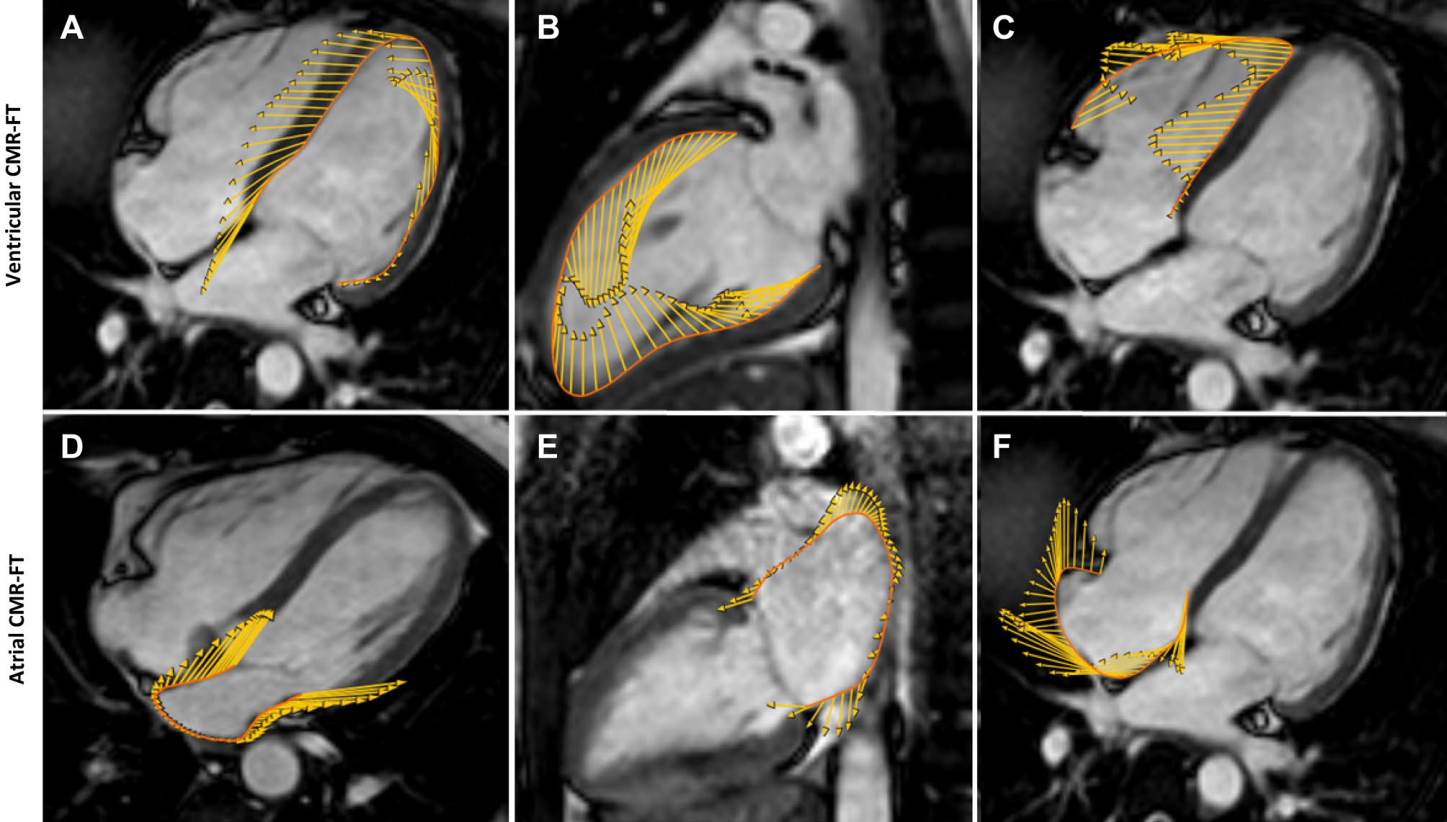
CMR-FT strain measurements in all four cardiac chambers in long axis orientation. The upper row (**A**–**C**) represents left and right ventricular strain, while the lower row (**D**–**F**) shows left and right atrial strain measurements. Note, length and orientation of arrows display relative extend and direction of deformation.

All CMR data analysis was performed by 2 trained observers blinded to all clinical information. All reported CMR values represent means of both observers. Cardiac volumes and functions were assessed using post-processing software (Medis Suite MR, QStrain 2.0.70.2, Leiden, Netherlands, Fig. 1). LV and RV volumes, LV mass and LV maximal wall thickness were obtained from cine short-axis. LA/RA volumetry were obtained from cine long-axis^20^. LVH was defined as maximum wall thickness (MWT) > 12 mm or increased indexed rendleft ventricular mass (LVMi) on CMR according to age- and sex-matched normal reference ranges in adults^16^. For most accurate CMR-FT measurements, endocardial and epicardial contours drawn on cine images were transferred to QStrain, where the tissue tracking algorithm was applied including an automated detection of myocardial borders throughout the complete cardiac cycle. Long-axis cine images were used to compute global longitudinal strain (GLS) and short-axis images were used to compute global circumferential (GCS) strain. Both orientations were used to compute global radial (GRS) strain and are presented separately. Long-axis RV, LA and RA contours were tracked from endocardium in long-axis slices^21^. Global longitudinal atrial strain (LA-GLS, RA-GLS) was further divided into the three atrial phasic functions^22^: LA/RA_Total_ strain (εs, the sum of passive and active strain, atrial reservoir function), LA/RA_Conduit_ strain (εe, passive strain, atrial conduit function), and LA/RA_Booster_ strain (εa, active strain, atrial contractile booster pump function). Global LA and LV longitudinal strain measurements were averaged in 2- and 4-chamber views. LA/RA maximum/minimum was defined at end-systole/end-diastole^12^. LGE was assessed semi-quantitatively^20^ on PSIR images using 5 standard deviations (SD) above the mean remote myocardium^23^ and presented in % of total LV mass.

### Statistical analysis

Normality of continuous data was tested using Shapiro–Wilk test. Categorical data are presented as counts/percentages and continuous variables as means/standard deviation. Mann–Whitney U test or Student’s t test was used for two patients’ group comparisons, where appropriate. Three patients’ group were compared by one-way analysis of variance (ANOVA) for normally distributed parameters or Kruskal–Wallis test otherwise with post hoc Tukey–Kramer test and Bonferroni correction for multiple comparisons (*p* < 0.017 for testing between three independent groups; *p* < 0.01 for testing between six independent groups). Fischer exact and χ2 tests were used for comparing proportions. Inter-observer agreement was assessed by intra-class correlation coefficients (ICC) and defined as poor (ICC < 0.4), good (ICC = 0.4–0.75), or excellent (ICC > 0.75) agreement^24^. Abnormally low native T1 values were defined as T1 < 996 ms, i.e., 2SDs below the mean of the controls of 1036 ± 20 ms. Diagnostic accuracy of correctly identified FD patients and FD patients with normal septal T1 times was calculated by Receiver-operating characteristic (ROC) analyses. Optimal cut-offs were obtained by the Youden index. Areas under the curves (AUCs) were compared by the DeLong method. Statistical significance was defined as a two-tailed value of *p* < 0.05.

## Results

### FD patients and controls

Clinical and CMR parameters are presented in Table 1. Lyso-Gb3, hs Troponin T, and NT-proBNP values were higher in cFD compared to vFD and controls (*p* < 0.05 for all calculations). Only 20 cFD and none of the vFD received ERT at the timepoint of the CMR study (Table 1). Patients on ERT with probably more advanced disease stage and higher amount of myocardial sphingolipid storage had significantly lower native T1 times and significantly higher Lyso-Gb3 blood values compared to ERT-naïve patients (S2 Supplement). CFD had significantly higher maximum LV wall thickness, LV mass index (LVMi) and LV stroke volume index (LVSVi) compared to vFD and controls (Table 1). Presence of LGE was much more common in cFD compared to vFD (Table 1, S3, supplement).

**Table 1:** Clinical and CMR-based characteristics of classical and variant FD patients and controls.

	Classical FD n = 37	Variant FD n = 14	Controls n = 14	*p*-value
Clinical parameters				
Age, y	42 ± 11	42 ± 13	48 ± 8	0.13
Males, n (%)	17 (46)	2 (14)	7 (50)	0.08
BSA, m^2^	1.8 ± 0.2	1.8 ± 0.2	1.9 ± 0.2	0.22
Heart rate, bpm	64 ± 13	74 ± 10	68 ± 11	0.06
NYHA I	24 (65)	13 (93)	**–**	0.08
NYHA II	12 (32)	1 (7)	**–**	0.08
NYHA III	1 (3)	0 (0)	**–**	1.0
NYHA IV	0 (0)	0 (0)	**–**	**–**
Diastolic Dysfunction III^	4 (11)	2 (14)	–	0.66
Holter abnormalities°	11 (30)	1 (7)	**–**	0.14
Atrial fibrillation	1 (3)	0 (0)	**–**	1.0
AP/Syncope	7 (19)	1 (7)	**–**	0.42
Lyso-GB3, ng/ml	16 ± 16	1 ± 1	**–**	** < 0.001**
Troponin T, ng/L	16 ± 20*	4 ± 3	2 ± 1	**0.007**
NT-proBNP, pg/ml	333 ± 306*	119 ± 106	55 ± 36	**0.006**
ERT, n (%)	20 (54)	0 (0)	–	** < 0.001**
Basic CMR parameters				
LVH, n (%)	21 (57)	1 (7)	**–**	**0.001**
max. LVWT, mm	12 ± 4*	8 ± 2#	8 ± 1	** < 0.001**
LV Mass Index, g/m^2^	66 ± 29*	44 ± 18#	49 ± 25	**0.001**
LVEF, %	64 ± 10	60 ± 10	65 ± 7	0.51
LVEDVi, mL/m^2^	97 ± 21	86 ± 20	92 ± 19	0.10
LVESVi, mL/m^2^	40 ± 20	37 ± 18	33 ± 13	0.49
LVSVi, ml/m^2^	60 ± 9	49 ± 5#	59 ± 11	** < 0.001**
RVEF, %	56 ± 11	55 ± 7	55 ± 9	0.98
RVEDVi, mL/m^2^	92 ± 20	81 ± 16	87 ± 17	0.18
RVESVi, mL/m^2^	47 ± 12	39 ± 13	39 ± 10	0.06
RVSVi, mL/m^2^	46 ± 14	42 ± 7	48 ± 14	0.40
LAEDVi, mL/m^2^	19 ± 14	15 ± 7	15 ± 4	0.32
LAESVi, mL/m^2^	37 ± 14	36 ± 11	38 ± 6	0.89
RAEDVi, mL/m^2^	23 ± 7	21 ± 11	21 ± 9	0.73
RAESVi, mL/m^2^	40 ± 11	39 ± 16	38 ± 12	0.82
LGE, n (%)	16 (43)	1 (7)	**–**	**0.02**
5SD-LGE, %LV	7.4 ± 4	3.1	**–**	**–**
Septal native T1, ms	951 ± 51*	1035 ± 15#	1038 ± 20	** < 0.001**
CMR-FT				
LV-GLS, %	− 20 ± 4*	− 21 ± 3	− 24 ± 2	**0.003**
LV-GCS, %	− 20 ± 4*	− 22 ± 2	− 25 ± 4	**0.002**
LV-GRS, %	106 ± 30	100 ± 29	106 ± 28	0.83
LA_Total_-GLS, %	29 ± 10*	36 ± 10	37 ± 5	**0.008**
LA_Conduit_-GLS, %	15 ± 10*	22 ± 8	24 ± 5	**0.004**
LA_Booster_-GLS, %	14 ± 6	13 ± 10	13 ± 5	0.99
RV-GLS, %	− 27 ± 6	− 30 ± 5	− 31 ± 6	0.08
RA_Total_-GLS, %	30 ± 8	31 ± 7	32 ± 4	0.56
RA_Conduit_-GLS, %	15 ± 7	17 ± 10	17 ± 7	0.63
RA_Booster_-GLS, %	15 ± 6	15 ± 7	16 ± 7	0.84

Numbers are mean ± SD for continuous and n (%) for categorical data.

Bold numbers represent statistical significance.

*p < 0.017 versus Controls, # *p* < 0.017 versus classical FD, °VES, disturbance of repolarisation, pauses, nsVT, SVES, ^E/A ≥ 2. In one patient with atrial fibrillation the calculation of the E/A ratio was not possible due to the missing A wave.

AP: Angina pectoris, BSA: Body surface area, CMR-FT: Cardiac Magnetic Resonance Feature Tracking, ERT: Enzyme replacement therapy, FD: Fabry disease, LA/RAEDVi: Left atrial/Right atrial end-diastolic volume index, LA/RAESVi: Left atrial/Right atrial end-systolic volume index, LA_Booster_-GLS: Left atrial booster global longitudinal strain, LA_Conduit_-GLS: Left atrial conduit global longitudinal strain, LA_Total_-GLS: Left atrial total global longitudinal strain, LGE: Late gadolinium enhancement, LVEF: Left ventricular ejection fraction, LV/RVEDVi: Left ventricular/right ventricular end-diastolic volume index, LV/RVESVi: Left ventricular/right ventricular end-systolic volume index, LV-GCS: Left ventricular global circumferential strain, LV/RV-GLS: Left ventricular/right ventricular global longitudinal strain, LV-GRS: Left ventricular global radial strain, LVH: Left ventricular hypertrophy, LVWT: Left ventricular wall thickness, SD: Standard deviation.

**Figure 2:**
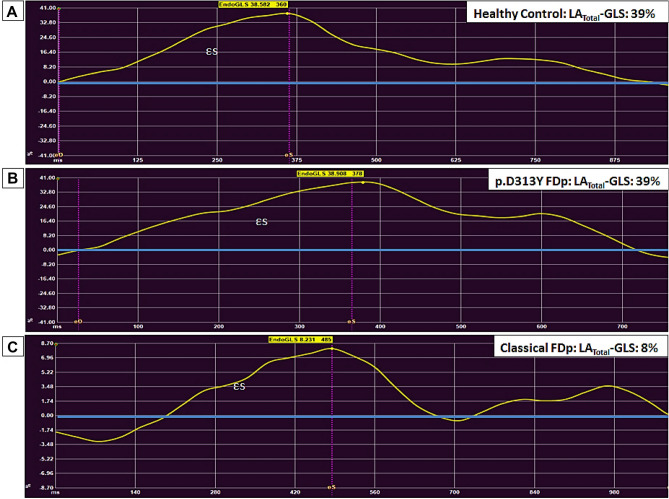
Comparison of LA_Total_-GLS (εs) between a healthy control (**A**), a p.D313Y FD variant (**B**) and a classical FD (**C**). Very similar LA_Total_-GLS values are obtained from p.D313Y FD variant and the healthy control compared to classical FD.

**Figure 3:**
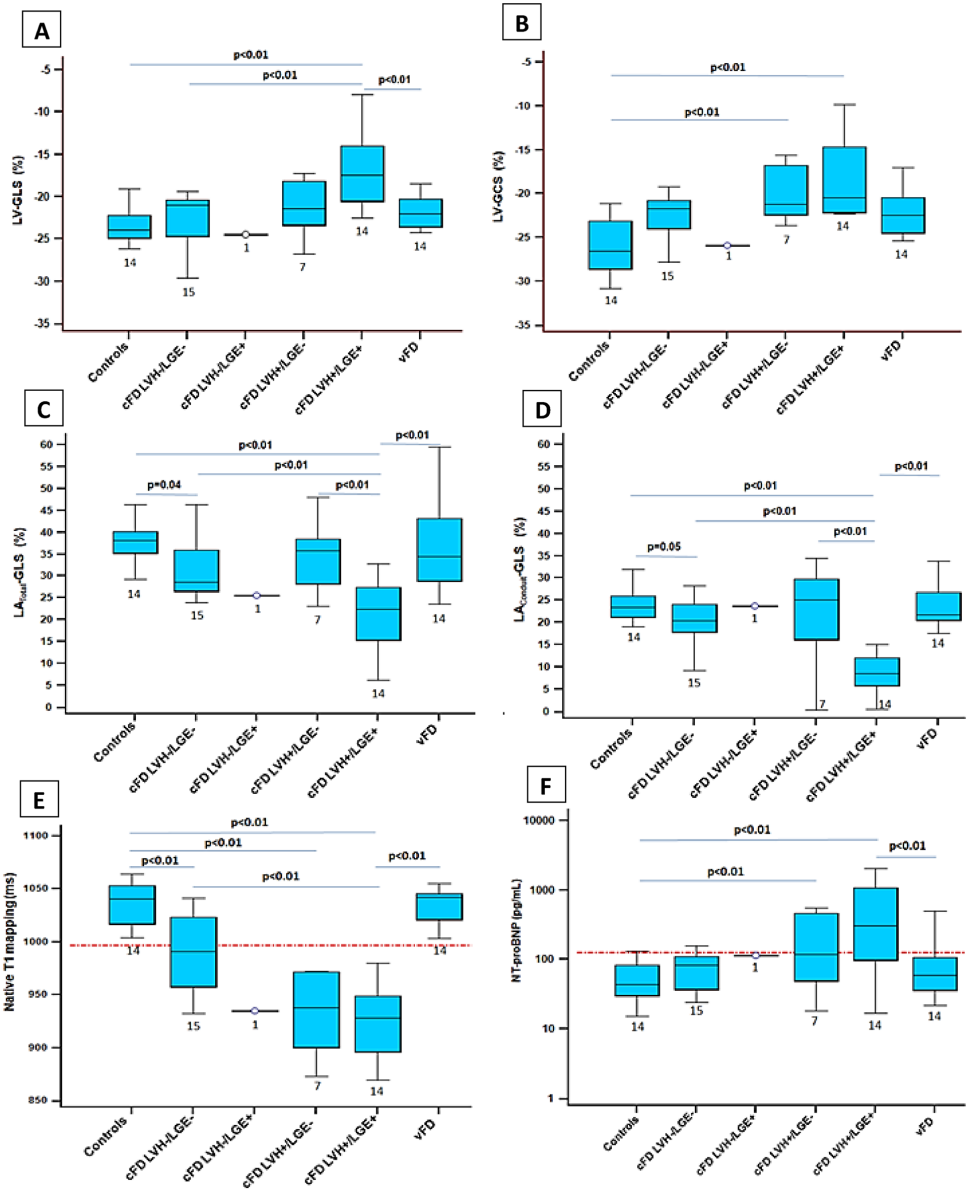
Impact of late gadolinium enhancement (LGE) and left ventricular hypertrophy (LVH) on CMR-FT strain, T1 mapping and NT-proBNP in cFD patients in comparison to vFD patients and controls. LV-GLS (**A**), LV-GCS (**B**), LA_Total_-GLS (**C**), LA_Conduit_-GLS (**D**) CMR-FT strain values, native T1 mapping (**E**), and NT-proBNP values (**F**) are presented in cFD patients, vFD patients and controls. Trends and significant *p*-values are provided in the graphs. The numbers of patients of each subgroup are presented below the box-plots. Dashed horizontal lines in (**E**) and (**F**) represent cutoff values for abnormality (T1 mapping: 996 ms; NT-proBNP:125 pg/ml).

Septal native T1 was significantly lower in cFD versus vFD and controls (Table 1). Significantly lower strain values (LV-GLS, LV-GCS, LA_Total_-GLS and LA_Conduit_-GLS) were found in cFD compared to controls (*p* < 0.05 for all, Table 2, Fig. 2). All parameters were comparable in vFD and controls (Table 1, Fig. 3).

**Table 2:** Sex-specific comparison of native T1 and CMR-FT in classical FD patients and controls.

	Classical FD males n = 17	Controls males n = 7	*p*-value	Classical FD females n = 20	Controls females n = 7	*p*-value
Age, y	40 ± 13	48 ± 7	0.13	43 ± 12	50 ± 11	0.19
LV Mass index, g/m^2^	83 ± 32	50 ± 9	** < 0.001**	51 ± 15	43 ± 22	0.26 0.001*
LVEF, %	68 ± 12	63 ± 4	0.25	72 ± 8	67 ± 5	0.17
LGE, n (%)	10 (59)	–	–	5 (25)	–	0.03*
5SD-LGE, %LV	9.3 ± 4	–	–	4.8 ± 3	–	0.04*
Septal native T1, ms	931 ± 54	1040 ± 14	** < 0.001**	971 ± 42	1029 ± 15	**0.006**
LV-GLS, %	−19 ± 4	−24 ± 2	**0.006**	− 22 ± 4	−24 ± 3	0.13 0.03*
LV-GCS, %	−19 ± 4	−25 ± 4	**0.002**	− 22 ± 4	−27 ± 2	0.01 0.03*
LA_Total_-GLS, %	28 ± 10	38 ± 7	**0.03**	28 ± 8	37 ± 6	**0.02**
LA_Conduit_-GLS, %	15 ± 12	26 ± 7	**0.03**	15 ± 9	22 ± 4	**0.04**
LA_Booster_-GLS, %	13 ± 6	12 ± 4	0.65	14 ± 7	15 ± 7	0.84

Numbers are mean ± SD for continuous and n for categorical data.

*Comparison between classical FD males and classical FD females.

Bold numbers represent statistical significance.

FD: Fabry disease, LA_Booster_-GLS: Left atrial booster global longitudinal strain, LA_Conduit_-GLS: Left atrial conduit global longitudinal strain, LA_Total_-GLS: Left atrial total global longitudinal strain, LGE: Late gadolinium enhancement, LVEF: Left ventricular ejection fraction, LV-GCS: Left ventricular global circumferential strain, LV-GLS: Left ventricular global longitudinal strain.

In male cFD native T1, LV-GLS, LV-GCS and LVMi were significantly altered with more pronounced difference compared to controls in contrast to female cFD (Table 2). LV-GLS and LVMi were comparable between female cFD and female controls (LV-GLS: *p* = 0.13; LVMi: *p* = 0.26). However, LV-GLS, LVMi, and the frequency and amount of LGE were significantly different in male compared to female cFD (LV-GLS: −19 ± 4 vs. −22 ± 4%, *p* = 0.03; LVMi: 83 ± 32 g/m^2^ vs. 51 ± 15 g/m^2^, *p* = 0.001; LGE frequency: 59% vs. 25%, *p* = 0.03; LGE amount: 9.3 ± 4%LV vs. 4.8 ± 3%LV, *p* = 0.04, Table 2). Native T1 mapping and LV-GCS values were sex-specific with significantly more reduced values found in male versus female cFD (native T1 mapping: 931 ± 54 ms vs. 971 ± 42 ms, *p* = 0.02; LV-GCS:−19 ± 4 vs. −22 ± 4%, *p* = 0.03), whereas all left atrial strain parameters didn’t show any sex-specific difference (LA_Total_-GLS (*p* = 0.99), LA_Conduit_-GLS (*p* = 0.81), LA_Booster_-GLS (*p* = 0.72)).

### Classical FD patients with (LVH +) and without left ventricular hypertrophy (LVH-)

CFD patients with left ventricular hypertrophy (cFD LVH +) were predominantly males (67%) and had significantly more Holter abnormalities like supraventricular/ventricular extrasystoles (SVES/VES), non-sustained ventricular tachycardia (nsVT), increased Lyso-Gb3 (21 ± 20 ng/ml vs. 9 ± 16 ng/ml), hs Troponin T (25 ± 24 ng/l vs. 5 ± 5 ng/l), NT-proBNP (526 ± 562 pg/ml vs. 80 ± 43 pg/ml) and higher prevalence of LGE (67% vs. 6%) compared to non-hypertrophied FD patients (cFD LVH-) (*p* < 0.05 for all, Table 3). Significantly reduced T1 times (931 ± 47 ms vs. 1037 ± 20 ms) and strain values for LV-GLS (−20 ± 5 vs. −24 ± 3%), LV-GCS (−20 ± 5 vs. −26 ± 3%), LA_Total_-GLS (26 ± 11 vs. 38 ± 5%) and LA_Conduit_-GLS (13 ± 10 vs. 24 ± 4%) were observed in the cFD LVH + cohort compared to controls (*p* < 0.017 for all, Table 3).

**Table 3:** Clinical and CMR-based characteristics of classical FD patients with (cFD LVH +) and without left ventricular hypertrophy (cFD LVH-) compared to controls.

	Classical FD LVH + n = 21	Classical FD LVH-n = 16	Controls n = 14	*p*-value
Age, y	45 ± 12	37 ± 12*	48 ± 8	**0.02**
Males, n (%)	14 (67)	3 (19)	7 (50)	**0.01**
Holter abnormalities°	9 (43)	1 (6)	–	**0.01**
Symptoms ‘	5 (24)	2 (13)	–	0.38
Lyso-GB3, ng/ml	21 ± 20	9 ± 16	–	**0.002**
Troponin T, ng/L	25 ± 24*	5 ± 5	2 ± 1	** < 0.001**
NT-proBNP, pg/ml	526 ± 562*	80 ± 43#	56 ± 37	**0.002**
Diastolic dysfunction III^	3 (14)	1 (6)	–	0.44
ERT, n (%)	13 (62)	7 (44)	–	0.27
LGE, n	14 (67)	1 (6)	–	** < 0.001**
5SD-LGE, %LV	7.5 ± 4.2	6.2	–	–
LV Mass Index, g/m^2^	80 ± 31*	47 ± 10#	50 ± 25	** < 0.001**
Septal native T1, ms	931 ± 47*	986 ± 39*#	1037 ± 20	** < 0.001**
LV-GLS, %	− 20 ± 5*	− 23 ± 3	− 24 ± 3	**0.005**
LV-GCS, %	− 20 ± 5*	− 22 ± 3	− 26 ± 3	**0.001**
LA_Total_-GLS, %	26 ± 11*	32 ± 8	38 ± 5	**0.001**
LA_Conduit_-GLS, %	13 ± 10*	18 ± 10	24 ± 4	**0.002**
LA_Booster_-GLS, %	14 ± 7	14 ± 6	13 ± 5	0.98

Numbers are mean ± SD for continuous and n (%) for categorical data.

Bold numbers represent statistical significance.

*p < 0.017 versus controls, # *p* < 0.017 versus classical FD LVH + , °including supraventricular and ventricular extrasystoles (SVES and VES), non-sustained ventricular tachycardia (nsVT), disturbances of repolarisation and pauses, ‘angina pectoris, syncope, ^E/A ≥ 2.

ERT: Enzyme replacement therapy, FD: Fabry disease, Gb3: Globotriaosylceramide, LA_Booster_-GLS: Left atrial booster global longitudinal strain, LA_Conduit_-GLS: Left atrial conduit global longitudinal strain, LA_Total_-GLS: Left atrial total global longitudinal strain, LGE: Late gadolinium enhancement, LV-GCS: Left ventricular global circumferential strain, LV-GLS: Left ventricular global longitudinal strain, SD: Standard deviation.

In hypertrophied cFD patients, LV-GCS was the only strain parameter, which was significantly reduced compared to controls independent of the presence of LGE (−18 ± 4% (cFD LVH + /LGE +) vs. −20 ± 3% (cFD LVH + /LGE-) vs. −26 ± 3% (Controls), *p* < 0.01, Fig. 3B). LA_Total_-GLS and LA_Conduit_-GLS showed a trend towards significant difference between non-hypertrophied cFD patients without LGE (LVH-/LGE-) and controls (32 ± 8%(LA_Total_-GLS) vs. 38 ± 5%(Controls), *p* = 0.04; 21 ± 5% (LA_Conduit_-GLS) vs. 24 ± 4% (Controls), *p* = 0.05, Fig. 3C,D). Only septal native T1 times were significantly lower in non-hypertrophied cFD patients without LGE (LVH-/LGE-) compared to controls (990 ± 37 ms vs. 1037 ± 20 ms, *p* < 0.01, Fig. 3E).

### Classical FD patients with normal septal T1 values

Six young female cFD (36 ± 10 years) had normal septal T1 values > 996 ms with minimally increased Lyso-Gb3 of 2.2 ± 1.1 (norm: < 0.9 ng/ml), normal other blood sample results (Troponin T < 14 pg/mL, NT-proBNP < 125 pg/ml), without left ventricular hypertrophy (LVH-), diastolic dysfunction (E/A > 2), atrial fibrillation or myocardial fibrosis (LGE-). However, compared with female controls, significantly larger left atrial volumes (LAESVi: 37 ± 4 ml/m^2^ vs. 31 ± 6 ml/m^2^, *p* = 0.03, lower LV-GCS (−22 ± 3 vs. −27 ± 2%, *p* = 0.02) and LA_Total_-GLS values were observed (29 ± 6 vs. 37 ± 4%, *p* = 0.02, Table 4).

**Table 4:** Clinical and CMR characteristics of classical FD patients with normal native T1 times compared to female controls.

	Classical FD (normal native T1) n = 6	Controls Females n = 7	*p*-value
Age, y	36 ± 10	50 ± 11	0.10
Males, n (%)	0 (0)	0 (0)	–
Lyso-GB3, ng/ml	2.2 ± 1.1	–	–
Troponin T, ng/L	3 ± 1	2 ± 1	0.33
NT-proBNP, pg/ml	95 ± 41	71 ± 41	0.31
Diastolic dysfunction III^	0 (0)	–	–
LV Mass index, g/m^2^	39 ± 6	43 ± 22	0.70
LGE, n	0 (0)	–	–
Septal native T1, ms	1021 ± 15	1029 ± 15	0.35
LAEDVi, mL/m^2^	20 ± 4	14 ± 5	**0.04**
LAESVi, mL/m^2^	37 ± 4	31 ± 6	**0.03**
LV-GLS, %	− 23 ± 4	− 24 ± 3	0.63
LV-GCS, %	− 22 ± 3	− 27 ± 2	**0.02**
LA_Total_-GLS, %	29 ± 6	37 ± 4	**0.02**
LA_Conduit_-GLS, %	17 ± 7	23 ± 4	0.09
LA_Booster_-GLS, %	12 ± 4	15 ± 7	0.46

Numbers are mean ± SD for continuous and n (%) for categorical data.

Bold numbers represent statistical significance.

^E/A ≥ 2.

FD: Fabry disease, Gb3: Globotriaosylceramide, LAEDVi: Left atrial/Right atrial end-diastolic volume index, LAESVi: Left atrial/Right atrial end-systolic volume index, LA_Booster_-GLS: Left atrial booster global longitudinal strain, LA_Conduit_-GLS: Left atrial conduit global longitudinal strain, LA_Total_-GLS: Left atrial total global longitudinal strain, LGE: Late gadolinium enhancement, LV-GCS: Left ventricular global circumferential strain, LV-GLS: Left ventricular global longitudinal strain.

### Identification of patients with classical Fabry disease

**Figure 4:**
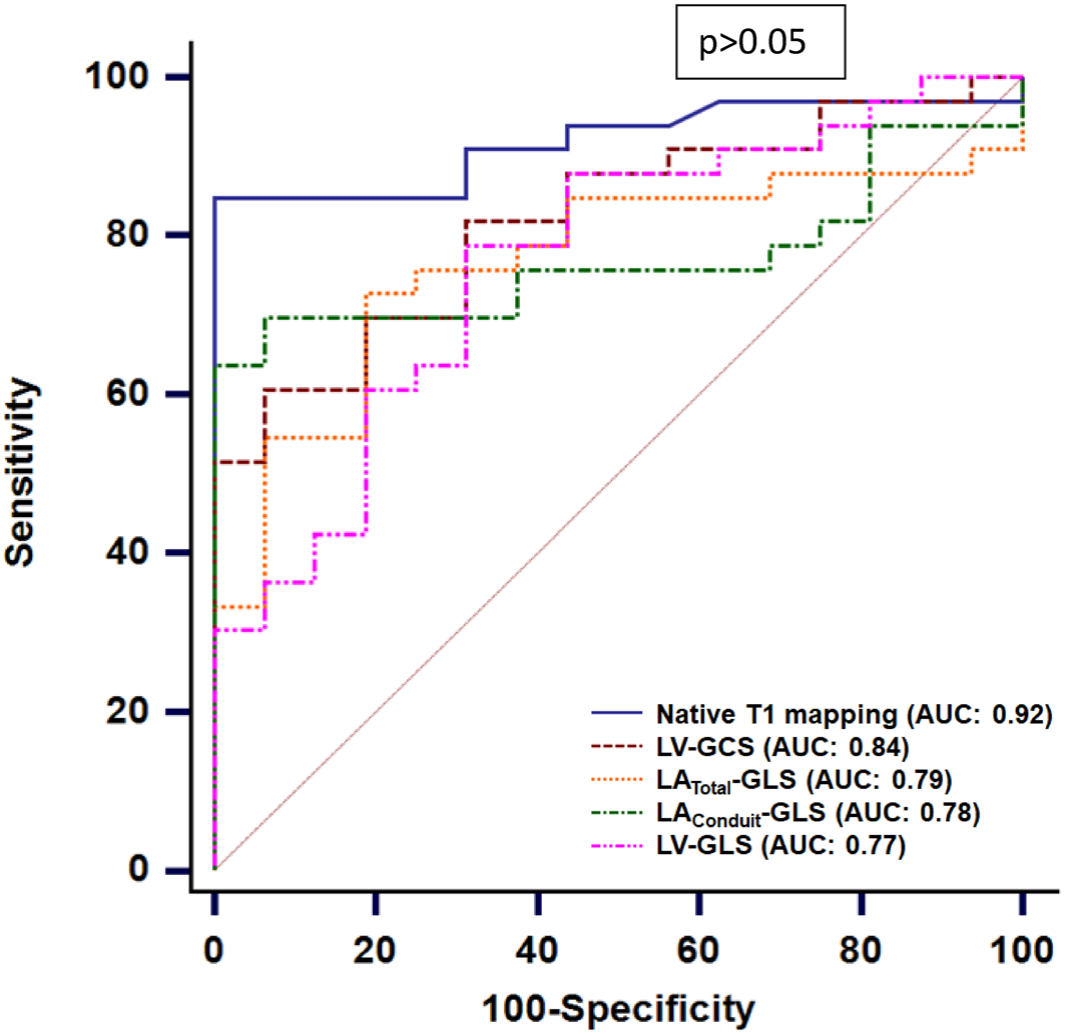
Pairwise comparison of ROC curves to identify classical FD patients. The ROC curves of left atrial (LA_Total_-GLS, LA_Conduit_-GLS) and left ventricular strain values (LV-GLS, LV-GCS) and native T1 mapping are presented with corresponding AUC values. Note, that no significant difference between the AUC curves was noticed (all *p* > 0.05).

The diagnostic yield of native T1 mapping and strain parameters to identify cFD was assessed by ROC analyses among the subgroup of 51 patients (37 cFD patients and 14 controls). Native T1 (cut-off: < 995 ms) had the highest AUC of 0.92 (95%CI: 0.81–0.98) for cFD discrimination, followed by LV-GCS (AUC: 0.84 (95%CI: 0.71–0.92), cut-off: > −21%) and LA_Total_-GLS (AUC: 0.79 (95%CI: 0.65–0.89), cut-off: < 33%, Table 5, Fig. 4).

**Table 5:** Diagnostic performance of left atrial, left ventricular strain values and native T1 mapping.

	CMR Parameter	AUC	Cut-off	Sensitivity	Specificity	PPV	NPV	Accuracy	*p*-value
Classical FD n = 37	Native T1	0.92 (0.81–0.98)	< 995 ms	84 (69–92)	100 (81–100)	100 (81–100)	73 (52–87)	88 (77–95)	< 0.001*
	LV-GCS	0.84 (0.71–0.92)	> -21%	62 (46–76)	94 (72–99)	96 (80–99)	52 (34–69)	72 (58–82)	< 0.001
	LA_Total_-GLS	0.79 (0.65–0.89)	< 33%	76 (60–87)	81 (57–93)	90 (75–97)	59 (39–77)	77 (64–87)	< 0.001
	LA_Conduit_-GLS	0.78 (0.64–0.88)	≤ 17%	65 (49–78)	94 (72–99)	96 (80–99)	54 (36–70)	74 (60–84)	< 0.001
	LV-GLS	0.77 (0.63–0.87)	> -23%	81 (66–91)	69 (44–86)	86 (71–94)	61 (39–80)	77 (64–87)	< 0.001
Classical FD/ normal native T1 n = 6	LV-GCS	0.70 (0.41–0.91)	> -23%	67 (30–90)	88 (53–98)	80 (38–96)	78 (45–94)	78 (52–93)	0.24
	LA_Total_-GLS	0.88 (0.59–0.9)	< 28%	67 (30–90)	100 (68–100)	100 (51–100)	80 (49–94)	86 (59–97)	< 0.001

Numbers are percentages (%) and 95% confidence intervals or continuous data.

*p > 0.05 for all pairwise AUC comparisons.

FD: Fabry disease; LA_Conduit_-GLS:Left atrial conduit global longitudinal strain: LA_Total_-GLS: Left atrial total global longitudinal strain; LV-GCS: Left ventricular global circumferential strain; LV-GLS: Left ventricular global longitudinal strain.

The resulting accuracies included: 88% (95%CI: 77–95) for native T1 mapping, 77% (95%CI: 64–87) for LA_Total_-GLS and LV-GLS, 74% (95%CI: 60–84) for LA_Conduit_-GLS and 72% (95%CI: 58–82) for LV-GCS without significant differences among the accuracies (*p* > 0.05 for all, Table 5). LA_Total_-GLS (cutoff: < 28%) had the highest AUC of 0.88 (95%CI: 59–90) and an accuracy of 86% (95%CI: 59–97) to identify cFD with normal T1 times among the subgroup of 13 females (6 cFD patients with normal T1 times and 7 healthy controls, Table 5, S3, supplement).

### Inter-observer agreement of myocardial strain

The inter-observer agreement for all strain measurements ranged from good (RA-GRS ICC: 0.55 (95% CI 0.43–0.78) to excellent (LV-GCS ICC: 0.85 (95%CI 0.72–0.92), LA_Total_-GLS ICC: 0.88 (95%CI 0.80–0.94), S4, supplement).

## Discussion

This study evaluated clinical and CMR-based parameters of cFD and vFD carrying the p.D313Y and p.A143T variants compared to controls and assessed the diagnostic yield of multiparametric CMR using native T1 mapping and CMR-FT strain in four cardiac chambers.

We found reduced myocardial strain in the left ventricle (LV-GLS, LV-GCS) and left atrium (LA_Total_-GLS, LA_Conduit_-GLS) in cFD, which revealed non-inferior diagnostic yield to native T1 mapping in discriminating cFD from controls (Fig. 4). For identification of female cFD patients with normal T1 values and normal LV mass index LA_Total_-GLS was the best parameter with an accuracy of 86%. All imaging parameters were comparable between vFD and the controls.

Significantly reduced LV-GLS, LV-GCS, LA_Total_-GLS, and LA_Conduit_-GLS were observed in the complete cFD group, which was obviously driven by hypertrophied cFD (Table 3). However, native T1 times were remarkably reduced in all cFD irrespective of the presence of left ventricular hypertrophy (Table 3). Our observed LV-GLS and LA_Total_-GLS alterations are supported by speckle tracking^25,26^ and CMR-FT studies^14^. However, other studies reported either no significant LV-GLS reduction in FD^15^ or just a regional apical and basal decrease in longitudinal strain (LS)^27^. This discrepancy of study results might be explained by several effects.

The percentage of female cFD predominated in these two studies with 63%^15^ and 57%^27^, alike in the present study with 54%, which might have affected the total group results. Regional LS differences between the LV base and apex might have caused global LS changes after averaging all regional LS values to one peak GLS, as observed in our and in other studies^15,27^. This requires further comparison of regional and global longitudinal strain in the future.

Our data reveal a sex-specific variance in LV-GLS and LV-GCS with significantly reduced values in male compared to female cFD patients (−19 ± 4 vs. −22 ± 4%, *p* = 0.03 for both LV-GLS and LV-GCS). This finding was accompanied by significantly reduced native T1 times in male compared to female cFD patients (931 ± 54 ms vs. 971 ± 42 ms, *p* = 0.02). We suppose that the sex-specific differences of left ventricular strain parameters (LV-GLS, LV-GCS) in this study are most probably caused by three major factors:

1. The X-linked mode of inheritance and the consequent significant reduction in alpha-galactosidase A activity lead to higher intracellular sphingolipid accumulation in the myocardium in male cFD patients, indicated by significantly reduced native T1 times compared to female cFD patients, as previously described^13,28^. This increased myocardial sphingolipid storage caused a mechanical dysfunction that impaired left ventricular strain, as shown by Vijapurau et al.^14^.
2. Significantly increased LVMi was found in our male cFD patients compared to female cFD patients, which was shown to be an independent predictor of impaired myocardial deformation on multivariate linear regression analysis by the same scientific group^14^.
3. Higher LGE frequency and amount in our male cFD patients resulted in a stiffer left ventricle with impaired myocardial contractility^2^.

In cFD LVH + patients only LV-GCS was significantly reduced irrespective of LGE compared to controls (Fig. 3). This is plausible, as LV-GCS reduction represents subepicardial and transmural fiber dysfunction ^29^ and early FD cardiomyopathy is caused by interstitial myocardial fibrosis predominantly in the mid-myocardial wall^30^, which mostly contributes to circumferential contraction and strain^31^ before progression towards replacement fibrosis^30^ assessable by LGE^9^. We didn’t observe significantly reduced LV-GCS in cFD LVH-, which was supported by a recent CMR-FT study^15^ and another echocardiographic study^27^, where only the regional apical circumferential strain was significantly reduced in cFD LVH-^27^. Furthermore, our predominance of female cFD LVH- (81%) with significantly increased LV-GCS compared to male cFD LVH-, might have caused comparable results to controls.

Interestingly, left atrial function assessment by LA_Total_-GLS and LA_Conduit_-GLS showed a tendency towards significant difference between non-hypertrophied cFD patients without LGE (LVH-/LGE-) and controls. Moreover, a subgroup of six female non-hypertrophied cFD patients with normal native septal T1 times and without atrial fibrillation had significantly reduced LA_Total_-GLS and LV-GCS values and significantly enlarged left atrial volumes compared to female controls (Table 4). However, only LA_Total_-GLS showed statistically significant AUC values, reaching a high accuracy of 86% (Table 5). We hypothesize, that in cFD patients deposits of glycosphingolipids in LA may occur at an early stage independent of those in the LV, as proven by left atrial biopsy^32,33^. Further, speckle tracking studies^25,26,34^ described abnormal LA mechanics in cFD due to interstitial atrial fibrosis and reduced LA_Total_-GLS was shown to be associated with atrial fibrillation and stroke^26,32,34^. As direct atrial fibrosis quantification by T1 mapping is hardly possible due to thin left atrial walls, LA_Total_-GLS strain might be the best parameter for the assessment of LA dysfunction to improve risk stratification and prognosis of FD patients at an early stage of the disease.

Although in our small cohort no single strain parameter could reliably identify the cFD LVH- subgroup unlike native T1 mapping, the AUC of LV-GCS for identifying all cFD was closest to that of T1 mapping without reaching significant difference(0.92 vs. 0.84, *p* > 0.05, Fig. 4). Moreover, especially in cFD LVH- patients T1 values can be normal in up to 50%^13^, which indicate an early stage of FD^11,28^. Thus, these patients, who most commonly do not have LGE either^11,28^, might be underdiagnosed.

The identification of early stage of Fabry disease is especially crucial and challenging in females. Female FD patients often present without LVH^28,35^ and with smaller decrease of native T1 times compared to males^11,13^. Therefore, according to our results, additional measurements of LV-GCS and LA_Total_-GLS besides native T1 mapping and LGE using multiparametric CMR may provide stronger confidence in an accurate identification of Fabry patients. One practical approach may be to screen patients with clinical suspect of Fabry disease with native T1 mapping and LGE. When T1 times are normal and LGE is absent, LA_Total_-GLS might provide additional diagnostic and prognostic value beyond T1 mapping in Fabry disease.

When LVH is present and T1 values are decreased, LV-GCS might add certainty in Fabry diagnosis besides T1 mapping and irrespective of LGE presence. An accurate detection of cardiac involvement in Fabry disease at an early stage would allow for earlier administration of enzyme replacement therapy (ERT) to further improve its prognosis^8^. Lastly, LA_Total_-GLS could detect improved LA function as sign of good therapy response in FD patients on ERT at 1-year follow-up in a speckle-tracking study^34^. Hence, LA_Total_-GLS assessed by CMR-FT may also be a valuable follow-up parameter in FD patients on ERT besides T1 mapping^36^.

Finally, all aforementioned strain parameters as well as their prognostic values require further investigation by larger, prospective studies in future.

Unlike cFD^11,14^, the p.D313Y and p.A143T variants were not associated with any subclinical/manifest cardiac involvement by LGE, T1 mapping or CMR-FT in our study. The LGE of the single p.D313Y FD variant was in apical inferolateral location, atypical for cFD, which probably resulted from the patient’s arterial hypertension and/or diabetes mellitus, rather than from the p.D313Y variant. This is in line with a recently published systematic review of 35 studies on p.D313Y, which found a low frequency of clinical features specific for cFD and no intracellular Gb3 accumulation in heart biopsies in p.D313Y carriers^37^. Instead, most studies revealed an involvement of the central and peripheral nervous system caused by p.D313Y^6,37^ and p.A143T^38^ FD variants.

### Limitations

Our study had several limitations. First, due to the rarity of FD and the retrospective study design we included a small number of selected Fabry patients and controls, so that the diagnostic performance of the provided CMR-derived parameters should be interpreted with caution. Moreover, an assessment of T2 mapping and adjustments for the duration of ERT or underlying disease severity was not possible, as previously described^14,15^. Second, CMR imaging was performed at 1.5 T using a single post-processing vendor for volumetry, T1 mapping, and CMR-FT, so our results may not be applicable to another software analysis due to variety of feature tracking strain results among different vendors^39^. Third, strain rates and regional strain parameters weren’t assessed as they are instantaneous measures with unavoidable estimation errors and consecutively less accurate^12^ compared to strain values, which are time-integral parameters.

## Conclusion

Left ventricular (LV-GLS/LV-GCS) and left atrial (LA_Total_-GLS) CMR-FT can detect impaired cardiac mechanics of cFD besides native T1 mapping with LV-GCS providing the closest diagnostic yield to native T1 mapping. A sex-specific variability existed for LV-GLS/LV-GCS with significantly lower strain values in male cFD. LA_Total_-GLS might accurately distinguish between oligosymptomatic female cFD patients and female controls. The FD-associated variants p.D313Y and p.A143T did not reveal any cardiac involvement by LGE, T1 mapping or CMR-FT.

## Supplementary Information

Supplementary Information.

## Data Availability

On reasonable request, all data used and/or analyzed during the current study are available from the corresponding author.

## Abbreviations

ANOVA: Analysis of variance
AP: Angina pectoris
AUC: Area under the curve
BSA: Body surface area
CMR-FT: Cardiac Magnetic Resonance Feature Tracking
cFD: Classic Fabry disease
ERT: Enzyme replacement therapy
FD: Fabry disease
Gb3: Globotriaosylceramide
GLA: Alpha galactosidase A gene
ICC: Intra-class correlation coefficients
LA/RAEDVi: Left atrial/Right atrial end-diastolic volume index
LA/RAESVi: Left atrial/Right atrial end-systolic volume index
LA/RA_Booster_-GLS: Left atrial/Right atrial booster global longitudinal strain
LA/RA_Conduit_-GLS: Left atrial/Right atrial conduit global longitudinal strain
LA/RA_Total_-GLS: Left atrial/Right atrial total global longitudinal strain
LGE: Late gadolinium enhancement
LVEF: Left ventricular ejection fraction
LV/RVEDVi: Left ventricular/right ventricular end-diastolic volume index
LV/RVESVi: Left ventricular/right ventricular end-systolic volume index
LV-GCS: Left ventricular global circumferential strain
LV/RV-GLS: Left ventricular/right ventricular global longitudinal strain
LV-GRS: Left ventricular global radial strain
LVH: Left ventricular hypertrophy
LVMi: Left ventricular mass index
LVWT: Left ventricular wall thickness
MOLLI: Modified look-locker inversion recovery
MWT: Maximum wall thickness
NYHA: New York Heart Association
PSIR: Phase-sensitive inversion recovery
ROC: Receiver-operating characteristic analysis
SD: Standard deviation
vFD: Fabry disease associated variants
