# Supplementary material for: Comparison of classical Fabry and its p.D313Y and p.A143T variants by cardiac T1 mapping, LGE and feature tracking myocardial strain
Source: Sci Rep. 2023 Apr 10;13:5809. doi: 10.1038/s41598-023-32464-0 (PMC10086062; doi:10.1038/s41598-023-32464-0)
Supplement: Supplementary file 1 — Supplementary Information. [file 41598_2023_32464_MOESM1_ESM.docx]

**Supplemental material**

**S1:** GLA mutations in classical (cFD) and variant FD patients (vFD) and their treatment by ERT

|  | **GLA mutation** | **Number of patients with mutation** | **Number of patients on ERT** | **Fabry-specific clinical phenotype** |
| --- | --- | --- | --- | --- |
| **Classical FD** | c.1277delAA | 4 | 4 | + |
|  | c.717delAA | 2 | 2 | + |
|  | c.718delAA | 4 | 4 | + |
|  | p.A230_I232del | 2 | 1 | + |
|  | p.A389V | 1 | 1 | + |
|  | p.C94S | 1 | 1 | + |
|  | p.E341K | 2 | 1 | + |
|  | p.I384N | 2 | 1 | + |
|  | p.L89del | 2 | 0 | + |
|  | p.N215S* | 6 | 1 | + |
|  | p.N320I | 1 | 0 | + |
|  | p.P205T | 1 | 0 | + |
|  | p.Q327L | 2 | 2 | + |
|  | p.R112C | 1 | 0 | + |
|  | p.R227Q | 1 | 1 | + |
|  | p.R227X | 1 | 1 | + |
|  | p.S247P | 2 | 1 | + |
|  | not documented | 2 | 0 | + |
| **Total** |  | **37** | **20** |  |
|  |  |  |  |  |
|  |  |  |  | **Clinical phenotype** |
| **Variant FD** | p.D313Y | 10# | 0 | RBBB (1)  Burning pain in extremities (4)  Stroke (1)  Polyneuropathy (2) |
|  | p.A143T | 4 | 0 | Arterial hypertension and LVH (1)  Synkope (1)  RBBB (1)  Burning pain in extremities (1) |
| **Total** |  | **14** | **0** |  |

*cardiac variant, which causes classical cardiac FD manifestations

#5 patients had no clinical Fabry-specific phenotype, but were family members of patients with Fabry-specific phenotypes

RBBB: Right bundle branch block

**S2:** Comparison of clinical and CMR parameters between classical FD patients on and without ERT at the time of CMR performance

|  | **Classical FD on ERT**  **n=20** | **Classical FD without ERT**  **n=17** | **p-value** |
| --- | --- | --- | --- |
| **Clinical parameters** | | | |
| **Age, y** | 44±11 | 39±14 | 0.25 |
| **Males, n (%)** | 10 (50) | 7 (41) | 0.16 |
| **Lyso-GB3, ng/ml** | 30±28 | 7±7 | **0.003** |
| **Troponin T, ng/L** | 21±24 | 13±18 | 0.29 |
| **NT-proBNP, pg/ml** | 465±504 | 208±202 | 0.15 |
| **Basic CMR parameters** | | | |
| **LVH, n (%)** | 13 (65) | 8 (47)* | 0.27 |
| **max. LVWT, mm** | 13±4 | 11±4 | 0.15 |
| **LV Mass Index, g/m²** | 70±25 | 62±32 | 0.40 |
| **LVEF, %** | 65±8 | 63±12 | 0.58 |
| **LVEDVi, mL/m²** | 98±19 | 96±23 | 0.71 |
| **LVESVi, mL/m²** | 40±15 | 39±25 | 0.96 |
| **LVSVi, ml/m²** | 62±10 | 57±7 | 0.10 |
| **LAEDVi, mL/m²** | 19±11 | 19±17 | 0.92 |
| **LAESVi, mL/m²** | 37±11 | 37±17 | 0.87 |
| **LGE, n (%)** | 9 (45) | 7 (41) | 0.60 |
| **5SD-LGE, %LV** | 6±4 | 10±3 | 0.06 |
| **Septal native T1, ms** | 931±30 | 969±60 | **0.03** |
| **CMR-FT** | | | |
| **LV-GLS, %** | -21±4 | -20±5 | 0.55 |
| **LV-GCS, %** | -21±3 | -21±4 | 0.87 |
| **LV-GRS, %** | 104±27 | 110±33 | 0.51 |
| **LA_Total_-GLS, %** | 28±5 | 29±10 | 0.71 |
| **LA_Conduit_-GLS, %** | 15±5 | 16±8 | 0.47 |
| **LA_Booster_-GLS, %** | 14±5 | 15±6 | 0.78 |

*In 6 patients ERT was initiated after the CMR revealed left ventricular hypertrophy.


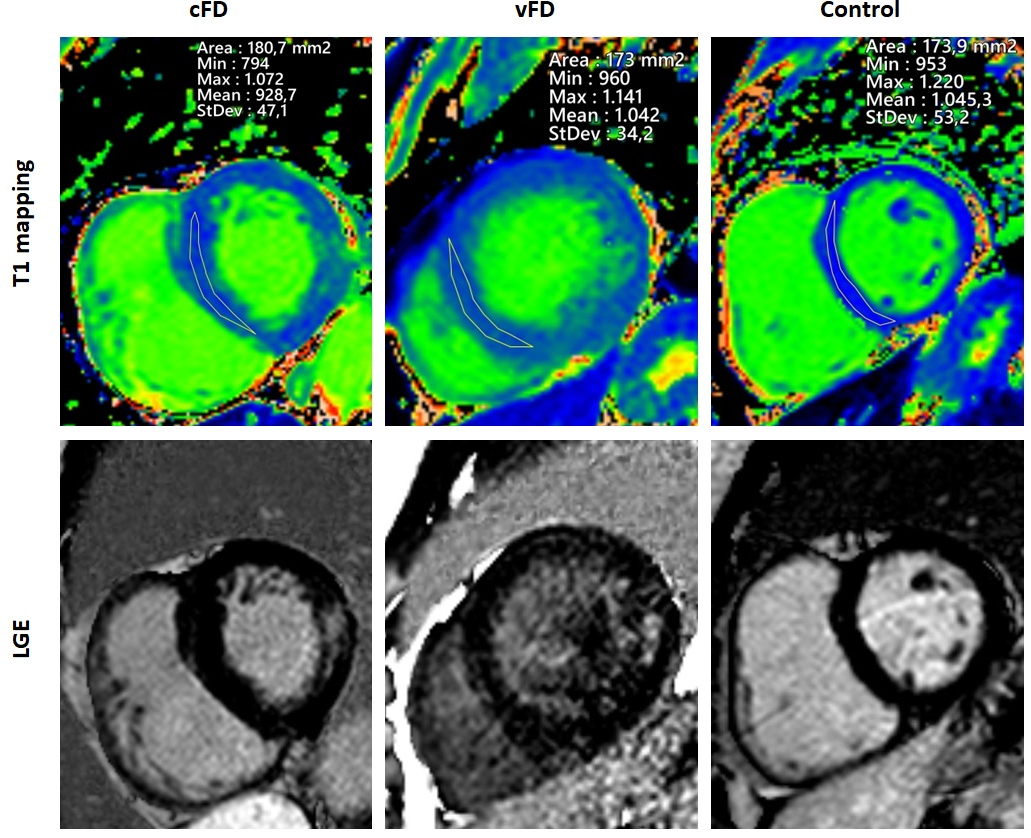


**A**

**D**

**B**

**C**

**EE**

**FE**

**S3:** **Comparison of septal nativeT1 mapping and LGE in two female patients with classical FD (cFD, A,D) and a p.D313Y** **variant FD (B,E) and a female control(C,F).** A 38-year old female cFD patient with moderate LVH (MWT: 17mm) showed significantly reduced septal native T1 times (929±47ms,**A**) and midmyocardial LGE in the basal inferolateral wall of the left ventricle (**D**, white arrows). A 44-year old female vFD patient with minor LVH (MWT: 13 mm), diastolic dysfunction III, diabetes mellitus, and arterial hypertension had normal septal native T1 times (1042±34ms, **B,E**), similar to the female 45-year-old control (1045±53ms, **C**), and midmyocardial LGE in the apical inferolateral wall of the left ventricle (**E**, white arrows). LVH: left ventricular hypertrophy; MWT: maximum wall thickness.

**
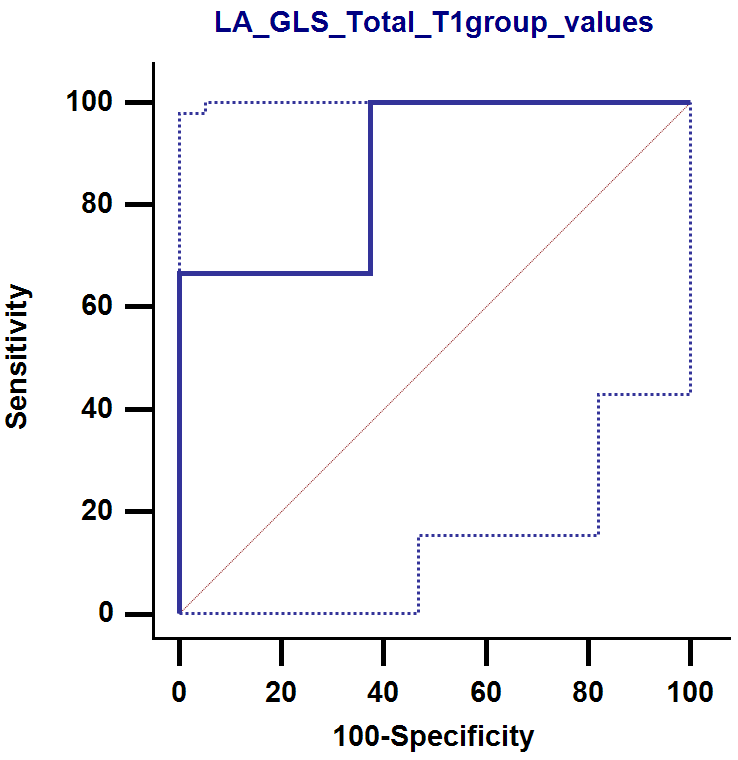
**

AUC: 0.88

**S4:** **ROC analysis of LA_Total_-GLS to identify classical FD patients with normal native T1 values.** The dashed lines represent the 95% confidence interval of the AUC of 0.88 (95%CI: 0.59-0.9).

**S5:** Interobserver agreement for different strain values

|  | **ICC (95%CI)** |
| --- | --- |
| **LV-GLS** | 0.80 (0.70-0.93) |
| **LV-GCS** | 0.85 (0.72-0.92) |
| **LV-GRS** | 0.57 (0.44-0.77) |
| **LA_Total_-GLS** | 0.88 (0.80-0.94) |
| **LA_Conduit_-GLS** | 0.73 (0.42-0.91) |
| **LA_Booster_-GLS** | 0.73 (0.42-0.91) |
| **LA-GCS** | 0.75 (0.56-0.87) |
| **LA-GRS** | 0.60 (0.40-0.72) |
| **RV-GLS** | 0.72 (0.43-0.86) |
| **RA_Total_-GLS** | 0.76 (0.55-0.88) |
| **RA_Conduit_-GLS** | 0.75 (0.56-0.87) |
| **RA_Booster_-GLS** | 0.75 (0.56-0.87) |
| **RA-GCS** | 0.79 (0.58-0.90) |
| **RA-GRS** | 0.55 (0.43-0.78) |
